# Supplementary material for: Clinicopathological Features of Non-Small Cell Lung Carcinoma with BRAF Mutation
Source: Curr Oncol. 2023 Nov 19;30(11):10019–32. doi: 10.3390/curroncol30110728 (PMC10670100; doi:10.3390/curroncol30110728)
Supplement: Supplementary file 1 [file curroncol-30-00728-s001.zip › curroncol-2678678-supplementary.pdf]

Supplementary Table S1. Pathological features of cases examined.

N	Age	Sex	Site	Material	Histotype	PD-L1	V600E (Y,N)	Aminoacid change	Co-mutations
1	67	M	hilar lymph node	cytological	adenoca	neg	N	D594N	CHD4
2	63	F	mediastinal lymph node	cytological	adenoca	high	Y	V600E	
3	65	M	bone	biopsy	adenoca	low	N	N581S	
4	74	M	lung	biopsy	adenoca	low	N	G596R	
5	74	M	lung	biopsy	adenoca	high	N	G466V	KRAS, PIK3CA
6	73	F	lung	biopsy	adenoca	low	Y	V600E	KRAS, MET
7	74	M	bone	biopsy	adenoca	low	N	G466A	FGFR3
8	85	M	lung	cytological	adenoca	high	N	D594F	KRAS, PAM2K1
9	83	F	lung	cytological	adenosquamous ca	high	N	N581S	
10	67	M	lung	cytological	adenoca	neg	N	D594N	
11	67	M	lung	biopsy	adenoca	neg	N	N581S	FGFR3
12	77	F	lung	biopsy	adenoca	low	N	L584F	KRAS
13	63	M	stomach (metastasis)	biopsy	adenoca	high	N	D594G	
14	78	M	bone	biopsy	adenoca	low	N	G469V	
15	79	M	pleura	biopsy	adenoca	high	Y	V600E	
16	76	M	hilar lymph node	cytological	adenoca	high	N	G469V	
17	52	M	pericardial effusion	cytological	adenoca	low	N	L597Q	
18	62	M	lung	biopsy	adenoca	neg	N	G466A	
19	71	M	pleura	biopsy	adenoca	low	N	G464V	KRAS
20	72	M	pleura	biopsy	adenoca	low	N	G469A	
21	84	M	lung	surgical	adenoca	low	Y	V600E	
22	45	F	lung	biopsy	adenoca	high	Y	V600E	AKT1, MTOR
23	58	F	mediastinal lymph node	cytological	adenoca	high	Y	V600E	
24	81	F	mediastinal lymph node	cytological	adenoca	low	N	N581S	
25	96	F	lung	cytological	adenoca		Y	V600E	
26	81	F	lung	cytological	adenoca	neg	Y	V600E	
27	64	M	liver (metastasis)	biopsy	adenoca	high	Y	V600E	IDH1
28	79	M	lung	biopsy	adenoca	high	Y	V600E	PIK3CA
29	65	F	lung	biopsy	adenoca	high	Y	V600E	
30	78	M	lung	surgical	adenoca	high	N	K601E	
31	67	M	lung	biopsy	adenoca	low	N	K601E	
32	79	F	mediastinal lymph node	cytological	adenoca	low	N	G469A	ESR1
33	79	M	lung	cytological	adenoca	high	Y	V600E	
34	75	M	lung	surgical	adenoca	low	Y	V600E	
35	68	M	lung	surgical	adenoca	neg	Y	V600E	PIK3CA
36	65	M	lung	biopsy	adenoca	low	Y	V600E	
37	68	F	lung	cytological	adenoca	low	Y	V600E	
38	55	M	lung	biopsy	adenoca	high	N	G464V	KRAS
39	55	F	lung	surgical	adenoca	low	N	G469V	
40	84	F	pleura	biopsy	adenoca	low	Y	V600E	
41	47	M	mediastinal lymph node	cytological	NOS	high	N	G469A	PIK3CA
42	73	F	lung	surgical	adenoca		N	D594N	
43	77	F	lung	cytological	adenoca		N	G469A	PIK3CA
44	55	F	hilar lymph node	surgical	adenoca		Y	V600E	
45	69	F	mediastinal lymph node	cytological	adenoca	high	Y	V600E	
46	68	F	lung	surgical	adenoca		N	G466A	

47	77	F	pleura	biopsy	adenoca	low	N	D594N	
48	74	M	mediastinal lymph node	cytological	adenoca	low	Y	V600E	
49	61	M	mediastinal lymph node	cytological	adenoca	high	N	G466A	
50	79	M	lung	biopsy	adenoca	low	N	E586G	EGFR
51	60	F	lung	biopsy	adenoca	neg	Y	V600E	IDH1
52	70	M	lung	biopsy	NOS		N	G258V	KRAS
53	72	M	lung	biopsy	squamous cell ca	high	N	V471F	
54	68	M	lung	biopsy	adenoca	low	Y	V600E	
55	73	F	lung	biopsy	adenoca	low	N	G466A	
56	83	F	lung	biopsy	adenoca	high	N	G466V	
57	80	M	bone	biopsy	adenoca	low	N	G466V	KRAS, EGFR
58	79	M	pleura	biopsy	adenoca	low	Y	V600E	
59	79	M	lung	surgical	adenoca		Y	V600E	
60	78	M	lung	biopsy	sarcomatoid ca	high	N	N581I	GNAQ

Legend: N Number, M male, F female, adenoca adenocarcinoma, ca carcinoma, NOS not otherwise specified, neg negative, Y yes, N no, TPS tumor proportion score). In PD-L1: negative TPS<1%; low TPS 1-49%, high TPS ≥50%.

Supplementary Table S2. Clinical stages and therapeutic regimens of cases examined. TKI therapy against BRAF is highlighted in red (alone and/or with CT: total N. 11 cases).

N	Age	Sex	Stage	Adjuvant therapy	Adjuvant regimen	First line therapy	First line regimen	Second line therapy	Second line regimen	Third line therapy	Third line regimen
**1**	67	M	IIIB			CT	carboplatinum, gemcitabine	none			
**2**	63	F	IV			none					
**3**	65	M	IV			CT	carboplatinum, pemetrexed	none			
**4**	74	M	IIIB			none					
**5**	74	M	IV			IT	pembrolizumab	CT	carboplatinum, pemetrexed	CT	paclitaxel
**6**	73	F	IV			CT-IT	carboplatinum, pemetrexed, pembrolizumab	TKI (BRAF)	dabrafenib, trametinib		
**7**	74	M	IV			CT-IT	carboplatinum, pemetrexed, pembrolizumab	none			
**8**	85	M	IV			IT	pembrolizumab	none			
**9**	83	F	IIIA			CT	carboplatinum, gemcitabine	nv			
**10**	67	M	IV			CT-IT	carboplatinum, pemetrexed, pembrolizumab	CT	carboplatinum, gemcitabine	none	
**11**	67	M	IV			CT-IT	carboplatinum pemetrexed, pembrolizumab	CT	docetaxel		
**12**	77	F	IV			none					
**13**	63	M	IV			IT	pembrolizumab	CT	carboplatinum, pemetrexed		
**14**	78	M	IV			none					
**15**	79	M	IV			TKI (BRAF)	dabrafenib, trametinib				
**16**	76	M	IV			IT	pembrolizumab	none			
**17**	52	M	IV			none					
**18**	62	M	IV			CT-IT	carboplatinum, pemetrexed,	CT	vinorelbine (oral)	none	

							pembrolizumab				
**19**	71	M	nv			nv					
**20**	72	M	nv			none					
**21**	84	M	nv (POR)			none					
**22**	45	F	nv			TKI (BRAF)	dabrafenib, trametinib	IT	pembrolizumab	CT	pemetrexed, cisplatinum
**23**	58	F	nv			IT	pembrolizumab	CT	carboplatinum gemcitabine	none	
**24**	81	F	nv			CT	gemcitabine	IT	atezolizumab		
**25**	96	F	nv			none					
**26**	81	F	IV			none					
**27**	64	M	IV			TKI (BRAF)	dabrafenib, trametinib				
**28**	79	M	IV			none					
**29**	65	F	IV			IT	atezolizumab				
**30**	78	M	nv (POR)			none					
**31**	67	M	nv			CT	carboplatinum, paclitaxel	IT	durvalumab		
**32**	79	F	IV			CT-IT	carboplatinum, pemetrexed, pembrolizumab	none			
**33**	79	M	IV			IT	pembrolizumab	none			
**34**	75	M	IIIB *			TKI (BRAF)	dabrafenib, trametinib	nv			
**35**	68	M	IIB	CT	cisplatinum, vinorelbine	none		nv			
**36**	65	M	IV			CT	carboplatinum, pemetrexed, pembrolizumab	TKI (BRAF)	dabrafeninb, trametinib		
**37**	68	F	IV			TKI (BRAF)	dabrafenib, trametinib	none			
**38**	55	M	IV			IT	pembrolizumab	none			
**39**	55	F	IIA*	CT		none					
**40**	84	F	IV			TKI (BRAF)	dabrafenib, trametinib				
**41**	47	M	IV			IT	pembrolizumab	none			
**42**	73	F	IIIA (POR)			none					
**43**	77	F	IV			CT	cisplatinum, pemetrexed	IT	nivolumab	CT	carboplatinum, gemcitabine
**44**	55	F	IIB*	CT	cisplatinum, gemcitabine	none					
**45**	69	F	IIIB			TKI (BRAF)	dabrafenib, trametinib				
**46**	68	F	IA* (POR)			none					
**47**	77	F	IV			CT-IT	carboplatinum, pemetrexed, pembrolizumab	CT	docetaxel (weekly)		
**48**	74	M	IV			IT	relatlimab, nivolumab, ipilimumab	CT	gemcitabine, carboplatinum	CT	vinorelbine
**49**	61	M	nv			nv					
**50**	79	M	IV			TKI (EGFR)	osimertinib	CT	gemcitabine	CT	vinorelbine

**51**	60	F	IIIC			CT	carboplatinum, paclitaxel	TKI (BRAF)	dabrafenib, trametinib		
**52**	70	M	nv			none					
**53**	72	M	nv			CT	gemcitabine, carboplatinum	IT	durvalumab		
**54**	68	M	nv			TKI (BRAF)	dabrafenib, trametinib	none			
**55**	73	F	nv			CT-IT	carboplatinum, pemetrexed, pembrolizumab				
**56**	83	F	nv			nv					
**57**	80	M	IV			nv					
**58**	79	M	IV			nv					
**59**	79	M	IA* (POR)			nv					
**60**	78	M	nv			IT	pembrolizumab				

Legend: N Number, CT chemotherapy, IT immunotherapy, TKIs tyrosine kinase inhibitors, nv not valuable (information not available), none no therapy (no therapy administered during FU), POR postoperative recurrence. * in these cases, the stage was based on the initial pathological diagnosis performed on the surgical specimen.

Supplementary Table S3. Data on OS and PFS.

N	Age	Sex	OS months	OS status	PFS months	PFS status
**1**	67	M	2	1	2	1
**2**	63	F	0	1	0	1
**3**	65	M	6	1	6	1
**4**	74	M	1	1	1	1
**5**	74	M	19	1	10	1
**6**	73	F	23	1	23	1
**7**	74	M	2	1	2	1
**8**	85	M	7	1	7	1
**9**	83	F	4	1	4	1
**10**	67	M	8	1	5	1
**11**	67	M	8	1	5	1
**12**	77	F	6	1	6	1
**13**	63	M	6	1	6	1
**14**	78	M	0	1	0	1
**15**	79	M	6	1	3	1
**16**	76	M	2	1	2	1
**17**	52	M	1	1	1	1
**18**	62	M	4	1	2	1
**19**	71	M	4	1	4	1
**20**	72	M	1	1	1	1
**21**	84	M	1	1	1	1
**22**	45	F	17	1	17	1
**23**	58	F	11	1	11	1
**24**	81	F	10	1	10	1
**25**	96	F	0	0*	0	0
**26**	81	F	21	1	21	1
**27**	64	M	6	1	6	1
**28**	79	M	1	1	1	1
**29**	65	F	14	1	14	1
**30**	78	M	0	1	0	1
**31**	67	M	9	1	9	1
**32**	79	F	25	0	20	1
**33**	79	M	47	0	47	0
**34**	75	M	10	0	10	0
**35**	68	M	42	0	42	0

**36**	65	M	33	0	3	1
**37**	68	F	33	0	33	0
**38**	55	M	30	0	30	0
**39**	55	F	18	0	18	0
**40**	84	F	13	0	13	0
**41**	47	M	42	0	23	1
**42**	73	F	18	0	18	0
**43**	77	F	10	0	10	0
**44**	55	F	7	0	7	0
**45**	69	F	8	0	8	0
**46**	68	F	2	0	2	0
**47**	77	F	13	1	3	1
**48**	74	M	49	0	45	1
**49**	61	M				
**50**	79	M				
**51**	60	F	46	0	1	1
**52**	70	M	2	1	2	1
**53**	72	M	27	1	27	1
**54**	68	M				
**55**	73	F	4	1	4	1
**56**	83	F	1	1	1	1
**57**	80	M				
**58**	79	M				
**59**	79	M	20	0	20	0
**60**	78	M				

Legend: N Number, OS overall survival, PFS progression-free survival. In OS Status and PFS Status: 1 means event and 0 censored. * OS Status was considered censored (died of other cause), since the patient had 96 years of age when deceased.

Supplementary Table S4. Summary of the Cox regression multivariate analysis.

	OS		PFS	
N. event	23		28	
N. censored	17		12	
N. total	40		40	
**Variables**	**HR (95% CI)**	**p**	**HR (95% CI)**	**p**
V600E (Y/N)	0.271 (0.103-0.717)	0.008	0.347 (0.145-0.831)	0.017
Stage (I,II,III,IV,V)	3.044 (1.003-9.238)	0.049	2.453 (0.983-6.121)	0.055
Therapy (TKI, IT, CT and CT-IT)	0.548 (0.356-0.842)	0.006	0.607 (0.417-0.883)	0.009

Legend: N number, OS overall survival, PFS progression-free survival, HR hazard ratio, CI confidence interval, Y yes, N no, CT chemotherapy, IT immunotherapy, TKI tyrosine kinase inhibitor.
